# Potential pathogenic and protective genotypes and phenotypes of vitamin D binding protein in multiple sclerosis

**DOI:** 10.3389/fneur.2025.1455779

**Published:** 2025-02-07

**Authors:** Suhail Al-Shammri, Arpita Chattopadhyay, Abu Salim Mustafa

**Affiliations:** ^1^Department of Medicine, College of Medicine, Kuwait University, Jabriya, Kuwait; ^2^Department of Medicine, Mubarak Al Kabeer Hospital, Ministry of Health, Jabriya, Kuwait; ^3^Department of Microbiology, College of Medicine, Kuwait University, Jabriya, Kuwait

**Keywords:** multiple sclerosis, vitamin D binding protein, polymorphism, genetic, vitamin D, Kuwait

## Abstract

**Background:**

The main carrier protein of vitamin D and its metabolites in plasma is vitamin D binding protein (VDBP) or group-specific component (Gc). Two single nucleotide polymorphisms, rs7041, and rs4588 in the *GC* gene result in three major VDBP/Gc genotypes, *GC1F* (c.1296T, c.1307C), *GC1S* (c.1296G, c.1307C), *GC2* (c.1296T, c.1307A), and phenotypes, Gc1F (p.432Asp, p.436Thr), Gc1S (p.432Glu, p.436Thr), and Gc2 (p.432Asp, p.436Lys). This study investigated frequencies of *GC* genotypes and phenotypes in Kuwaiti multiple sclerosis (MS) patients and healthy controls, and their associations with serum levels of 25 hydroxyvitamin D [25(OH)vitamin D] and VDBP.

**Methods:**

The genomic DNA was isolated from blood samples of drug-naïve MS patients (*N* = 151) and controls (*N* = 127). DNA regions covering the targeted mutations were amplified by PCR, sequenced by the Sanger method, and analyzed to determine *GC* genotypes and phenotypes. Serum 25(OH)vitamin D and VDBP levels were measured by enzyme immunoassay. SPSS used for statistical analyses. Differences between independent and related groups tested by Mann–Whitney *U* and Wilcoxon signed-rank tests respectively, Genotype and phenotype frequencies were calculated; *p* < 0.05 considered significant.

**Results:**

The study detected four Gc genotypes/phenotypes, namely *GC1F*/Gc1F (c.1296T, c.1307C/p.432Asp, p.436Thr), *GC1S*/Gc1S (c.1296G, c.1307C/p.432Glu, p.436Thr), *GC2*/Gc2 (c.1296T, c.1307A/p.432Asp, p.436Lys), and *GC3*/Gc3 (c.1296G; c.1307A/p.432Glu, p.436Lys) in both subjects. The frequencies of *GC3* genotype (control: 5.51%; patient: 28.48%) and Gc3-containing phenotypic groups (Gc1S/Gc3 + Gc2/Gc3 + Gc3/Gc3) were significantly higher in patients. Moreover, frequencies of *GC1F* genotype (control: 27.17%; patients: 5.30%) and Gc1F-containing phenotypic groups (Gc1F/Gc1F + Gc1S/Gc1F + Gc2/Gc1F) were higher in controls. Vitamin D levels were deficient in both groups. However, VDBP concentrations were significantly low in MS patients only.

**Conclusion:**

The VDBP/GC genotypes and phenotypes are associated with MS. Common genotype *GC1F* might be protective, and *GC3*, the novel variant found in MS patients appeared to be pathogenetic. Hypovitaminosis-D is prevalent in MS patients and controls.

## Introduction

Multiple sclerosis (MS) is a chronic inflammatory demyelinating disease of the central nervous system, influenced by a complex interplay of genetic predisposition and environmental triggers (1). While the precise cause of MS remains unclear, its prevalence varies significantly across geographic and ethnic groups (2). In Kuwait, the crude prevalence rate has shown a marked increase from 31.15/100,000 in 2000 to 85.05/100,000 in 2011, suggesting potential environmental contributions (3, 4). Studies indicated that genetic factors such as human leukocyte antigens (HLA) (5) and environmental influences like geographical locations (6), sun exposure (7–9), dietary intake (9), vitamin D levels (10), may play significant roles in MS risk (11).

Vitamin D and its carrier protein, vitamin D binding protein (VDBP), have been implicated in MS pathogenesis (9, 10, 12). The bioactive metabolite 1,25-hydroxyvitamin D is a secosteroid hormone that is synthesized in the skin on exposure to ultraviolet radiation (13). A collaborative study from US reported that the risk of developing MS decreases with increasing levels of serum 25 hydroxyvitamin D3 [25(OH) D3] (10). MS disease activity influenced by 25(OH) D3 status (14). Low levels of vitamin D are associated with accrual of disability, and increased relapse rate (15). Furthermore, vitamin D supplementation has been shown to ameliorate disease activity in the MS experimental model; experimental autoimmune encephalitis (16).

Proteomic analysis studies have explored the role of vitamin D binding protein (VDBP), also known as the group-specific component (Gc), in multiple sclerosis (MS) (17, 18). Synthesized in the liver, VDBP binds and transports vitamin D and its metabolites to target organs (19). Genetic variations in the VDBP gene, particularly two single nucleotide polymorphisms (SNPs), rs7041 and rs4588, lead to three key isoforms: Gc1 fast (Gc1F), Gc1 slow (GC1F), and Gc2 (20). These isoforms lead to three major VDBP/Gc genotypes (*GC*), namely *GC1F* (c.1296T, c.1307C), *GC1S* (c.1296G, c.1307C), and *GC2* (c.1296T, c.1307A); and phenotypes (Gc), namely Gc1F (p.432Asp, p.436Thr), Gc1S (p.432Glu, p.436Thr), and Gc2 (p.432Asp, p.436Lys) (21).

These isoforms influence vitamin D bioavailability, affecting genetic susceptibility to diseases (22). Proteomic research has identified lower VDBP levels in the cerebrospinal fluid of MS patients, especially during acute relapses (23). Genetic studies examining the VDBP gene’s role in MS have shown mixed results. Research from Japan, Canada, and Italy found no significant association between GC genotypes and MS risk, although these findings might not apply universally due to population specific VDBP isoform distributions (13, 24, 25). Interestingly, VDBP polymorphisms have been linked to other chronic immune-related diseases like asthma and diabetes (22). Recent studies have observed lower VDBP levels in MS patients compared to healthy individuals, though the role of GC genotypes in these variations remains unexplored (26, 27). These findings highlight the need for further investigation into VDBP polymorphisms and their interplay with vitamin D metabolism and MS susceptibility across diverse populations.

In this study, we investigated 1,112 alleles from 278 subjects (151 drug naïve Kuwaiti MS patients and 127 healthy controls) to explore the distribution of VDBP genotypes and phenotypes and their association with serum levels of 25-hydroxyvitamin D [25(OH)vitamin D] and VDBP. Each chromosome in a diploid somatic cell is made up of two chromatids, each of which contains a copy of an allele at each genetic location. A diploid cell contains 4 copies of an allele. We aim to determine whether these genetic variations contribute to MS susceptibility in the Kuwaiti population and their associations (if any) with the serum concentrations of 25(OH)vitamin D and VDBP.

## Methods

### Subjects

In this observational study, we recruited 151 drug naïve Kuwaiti patients diagnosed with MS and 127 healthy controls. Recruitment of subjects (both patients and controls) and data collection was done from October 2017 to October 2018. The sample calculation was done by unmatched case-control study following OpenEpi, Version 3 (28). Both groups of subjects were approximately frequency matched for age and sex (29). MS patients were recruited from the MS clinic of Mubarak Al Kabeer Hospital, Kuwait, and the control subjects were selected from healthy individuals among the Kuwaiti population. The MS patients were diagnosed according to the McDonald criteria (30) and followed up by our team of experienced neurologists at the national MS clinic at Mubarak Al-Kabeer Hospital in Kuwait. Type of MS was identified (30) and disability due to MS was measured using the expanded disability status scale (EDSS) (31). At the time of recruitment, none of the control subjects had any documented history of autoimmune, inflammatory, or neurologic diseases and had no family history of MS. Moreover, none of the patients were taking any disease-modifying drugs and they did not receive corticosteroids during the month prior to the evaluation. The female subjects (controls and patients) were not on estrogen supplementation.

All the participants signed a voluntary informed consent form, which was approved by the Ethics Committees of the Health Sciences Centre, Kuwait University approval number-VDR/EC/3073-June-6, 2017.

Both patients and controls were interviewed and requested to fill in a questionnaire that was designed to collect information like age, gender, body mass index (BMI), daily habits (including sun exposure, choice of routine outdoor dressing, routine diet, and outdoor physical activity, etc.), past medical history, and current and past medications (including dietary supplements). Like any other observational case controls study the potential for recall bias cannot be ruled out in this study.

### Biochemical analysis

Overnight fasting blood specimens were collected from all subjects and immediately centrifuged at 3,000 RPM for 15 min, and the serum samples were stored at −40°C until analyzed using commercially available kits. The enzyme immunoassay (EIA) was performed to estimate the levels of total 25(OH)vitamin D (Immune Diagnostic Systems, Bensheim, Germany) following the kit manufacturer’s protocols.

The procedure for estimating 25(OH)vitamin D in serum involves dilution of calibrators, controls, and samples with biotin labelled 25(OH)vitamin D. The diluted samples were incubated in highly specific sheep 25(OH)vitamin D antibody coated microtiter wells for 2 h at room temperature before aspiration and washing. Enzyme (horseradish peroxidase) labelled avidin, was added and binds selectively to complexed biotin and, following a further wash step, colour was developed using a chromogenic substrate (TMB). The absorbance of the stopped reaction mixtures was read in a microtiter plate reader, colour intensity developed being proportional to the concentration of 25-(OH)vitamin D. The intra-assay coefficients of variations (CVs) were 5.3 and 5.6% at 39.0 and 61.1 nmol/L, respectively. The inter-assay CVs were 4.6 and 6.4% at 40.3 and 72.0 nmol/L, respectively. A quantitative Sandwich Enzyme Immunoassay was conducted using the manufacturer’s procedure to estimate VDBP (Quantikine, R&D systems, Minneapolis, MN, United States). In brief, the procedure involved dilution of standard, control serum sample with calibrator diluent RD6-11 followed by incubation in highly specific monoclonal VDBP antibody coated microplate for 1 h at room temperature before aspiration and washing. After washing away any outbound substances, an enzyme linked monoclonal antibody specific for VDBP was added to the well, following a further wash step, a chromogenic substrate solution was added to the well and colour was developed. The absorbance of the stopped reaction mixtures was read in a microtiter plate reader, colour intensity developed being proportional to the concentration of VDBP bound in initial step. The intra-assay coefficients of variations (CVs) were 5.7 and 6.2% at 33.0 and 180 ng/mL, respectively. The inter-assay CVs were 5.1 and 7.4% at 52.9 and 164 ng/mL, respectively.

As per the recommendations of the clinical practice guidelines of the Endocrine Society Task Force (32), the cutoff points utilized for classification in this study were: serum 25(OH)vitamin D <50 nmol/L—vitamin D deficiency; <25 nmol/L—severe vitamin D deficiency (33), and inadequate or insufficient at 50–75 nmol/L (20–30 ng/mL) and sufficient or adequate at >75 nmol/L (>30 ng/mL) (34, 35).

### Isolation of genomic DNA

Genomic DNA was isolated from whole blood of MS patients and healthy controls using the DNA Blood Mini Kit (Qiagen, Hilden, Germany), following the manufacturer’s instructions. The quality and quantity of the isolated genomic DNA were determined using a low volume (2 μL sample) Epoch Microplate Spectrophotometer (BioTek Instruments, Inc., Winooski, VT, United States) by measuring the absorbance at 260 nm and 280 nm. DNA from these samples was used for determining *GC* genotypes. Conventional PCR method was used for amplification of the target DNA sample.

### Oligonucleotide primers for amplification of the GC target DNA

The forward (5-gatctcgaagaggcatgtttc-3′) and reverse (5′-gttgcctgtgttcacagactc-3′) primers were designed based on the genomic DNA sequence of human *GC* gene (GenBank: L10641.1, gene length = 55,136 nucleotides) using Primer Design Software of National Centre for Biotechnology Information (NCBI), Bethesda, MD, United States. These primers annealed at the sites in the *GC* gene, which encompassed the two specific mutation sites, i.e., G and C at the locations 35,706 and 35,717 in the *GC* gene, respectively (21).

### PCR amplification of target region

The forward (F) and reverse (R) primers were used in PCR amplification according to standard methods (21). In brief, PCRs were performed in 0.2 mL microtubes with a total volume of 50 μL containing 100 ng of genomic DNA, 250 μM of each dNTP, 10 mM tris-HCl (pH 8.3), 50 Mm KCl, 2 mM MgCl_2_, 2.5 units of *AmpliTaq* Gold^®^ DNA polymerase (Thermo Fisher Scientific, Waltham, MA, United States) and 25 pmol of each F and R primer. PCR cycles were performed with an initial denaturation step of 10 min at 95°C for the activation of Ampli-*Taq*Gold, followed by 30 cycles of 94°C for 30 s, 60°C for 30 s, and 72°C for 30 s with a final extension step of 72°C for 5 min. The expected size of PCR product (597 bp) was confirmed by agarose gel electrophoresis using 1.5% agarose gels (21, 36). DNA extracted from human subjects from the same population was used as positive control and mastermix without any DNA used as negative control.

### Sequencing of PCR products and analysis of data

The PCR products were sequenced according to Sanger’s method using an automated DNA Sequencing System (ABI 3130xl Genetic Analyzer, Thermo Fisher Scientific) (21). In brief, the purification of amplified products was performed by addition of 2 μL of ExoSAP-IT^®^ to 5 μL of the PCR product and incubated for 15 min at 37°C, followed by heating at 80°C for 15 min, which made the enzyme inactive. The purified DNA fragments (10 ng) were used in the cycle-sequencing reaction using a BigDye Terminator v1.3 Cycle Sequencing Kit (Thermo Fisher Scientific). The generated DNA fragments were purified using BigDye XTerminator^®^ Purification Kits (Thermo Fisher Scientific) and analyzed using an ABI 3130x Genetic Analyzer (Thermo Fisher Scientific). DNA sequence data of the PCR amplified fragments were analyzed for specific mutations rs7041 (G − T) and rs4588 (C − A) using the software in the Genetic Analyzer. Based on these results, the frequencies of *GC* genotypes and phenotypes were determined (36).

### Statistical analyses

Statistical analyses were carried out using Statistical Package for Social Sciences (SPSS) (IBM Corporation, Armonk, NY, United States 2013). Qualitative variables were described using numbers and percentages, while quantitative variables were obtained using medians (25th–75th percentile) and mean ± SD, as appropriate. Differences between two independent groups were tested with the Mann–Whitney *U* test. Differences between two related samples were tested with the Wilcoxon signed-rank test. The genotype and phenotype frequencies in MS patients and controls were calculated and the results were compared using Fisher’s exact test or chi square test (as appropriate). *p* < 0.05 was taken as significant. Missing variables are removed from the analysis.

## Results

### Study population

Initially, following the sample size calculated (28), a total of 188 potentially eligible patients and equal number of control subjects were included in the study, however 37 patients and 61 controls withdrawn from the study citing personal circumstances and excluded from analysis. The descriptive characteristics of rest the cohort are presented in Table 1. The subjects were young adults (mean age: 33.6 ± 10.1 years) and none of them reported to take any medications. The study population of Kuwaiti nationals comprised of three ethnicities, i.e., non-Bedouin Arabs (44.5%) Bedouin Arabs (42.2%) and Persians (13.3%). The patients were diagnosed having MS at early adulthood (mean age: 28.2 ± 9.3) with a duration of disease varying from 0–6 years. Most of the patients (86.1%, *N* = 130) were having relapsing remitting MS (RRMS), followed by secondary progressive (RP) (12.6%, *N* = 19) and primary progressive (PP) (1.3%, *N* = 2) MS (Table 1). None of the patients were reported to take immunomodulatory therapy. Disease severity measures by EDSS was 1.5 (0–3) [Quartile 1 (Q1)–Quartile 3 (Q3)] (Table 1).

**Table 1:** Demographics characteristics of healthy subjects and MS patients and clinical dada of MS patients.

Variables	All *N* = 278	Healthy subjects *N* = 127	MS patients *N* = 151
Gender, *N* (%)			
Male	104 (37.4)	45 (35.4)	59 (39.1)
Female	174 (62.6)	82 (64.6)	92 (60.9)
Ethnicity, *N* (%)			
Bedouins	98 (35.3)	54 (42.5)	44 (29.1)
Non-Bedouins	133 (47.8)	56 (44.1)	77 (51.0)
Persians	47 (16.9)	17 (13.4)	30 (19.9)
Age in years, mean ± SD	33.6 ± 10.1	33.7 ± 9.3	28.7 ± 5.5
BMI (kg/m^2^), mean ± SD	28.1 ± 5.6	33.5 ± 10.7	27.7 ± 5.7
Family history of MS, *N* (%)			
Negative	229 (82.4)	127 (100)	108 (71.5)
Positive	49 (17.6)	0	43 (28.5)
Clinical data of MS patients			
Age at diagnosis in years, mean ± SD	—	—	28.2 ± 9.3
Duration of MS in years, mean ± SD	—	—	5.3 ± 5.9
MS subtypes, *N* (%)			
Relapsing remitting MS	130 (86.1)		
Secondary progressive MS	19 (12.6)	—	—
Primary progressive MS	2 (1.3)	—	—
EDSS, median (Q1–Q3)	1.5 (0–3)	—	—

*N*, number of subjects; *N* (%), number of subjects (percentage); mean ± SD, mean ± standard deviation; BMI, bone mass index.

### Genotyping analysis

**Figure 1:**
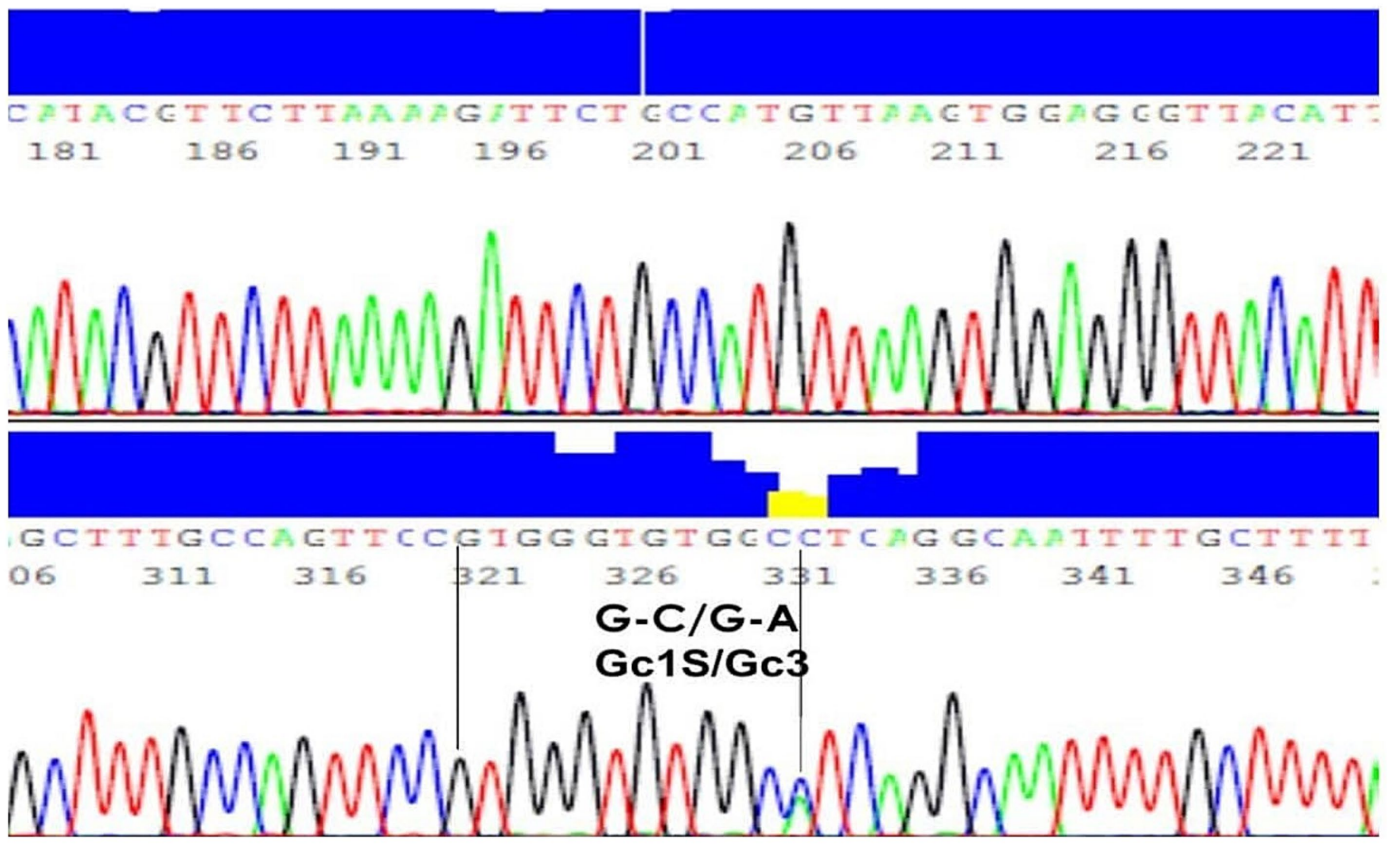
DNA sequence of a representative sample with genotype G-C/G-A and phenotype Gc1S/Gc3. A representative result of PCR amplification, followed by agarose gel electrophoresis, showed that the product of specific size (597 bp DNA) was amplified from the genomic DNA.

The results of PCR amplification, followed by agarose gel electrophoresis, showed that the product of specific size (597 bp DNA) was amplified from the genomic DNA of all samples. The sequencing of PCR amplified DNA was successful in all controls and patients, and the sequence analyses confirmed that the PCR products belonged to the *GC* gene. PCR amplification efficiency was 100%, sequencing read quality was highly efficient to provide successful sequencing of all samples included in this study. Furthermore, the sequence analyses identified the nucleotide bases present in the *GC* gene at the specific mutation sites, i.e., locations 35,706 (G to T) and 35,717 (C to A). A representative result is shown in Figure 1 (21).

### GC allelic subtypes

The overall analyses of DNA sequence data identified the existence of four different allelic subtypes of *GC*, namely *GC1S*, *GC1F*, *GC2* and *GC3* in the study population (Table 2). Among the total allelic subtypes in controls (*N* = 508 alleles in 121 healthy subjects) and patients (*N* = 604 alleles in 151 MS patients), *GC1S* was the predominant subtype present in almost equal frequencies in controls (*N* = 270, 53.15%) and patients (*N* = 306, 50.66%), *p* > 0.05 (Table 2). However, the frequency of *GC1F* allele was significantly higher in controls (*N* = 138, 27.17%) than in patients (*N* = 32, 5.3%) (*p* < 0.005) and the frequency of *GC3* was significantly higher in patients (*N* = 172, 28.48%) than in controls (*N* = 28, 5.51%) (*p* < 0.005). *GC2* frequencies were almost similar in controls (*N* = 72, 14.17%) and patients (*N* = 94, 15.56%), (*p* > 0.05). All *p*-values were generated using the Pearson chi-square test in Table 2.

**Table 2:** Types of *GC* alleles in healthy subjects and MS patients.

*GC* alleles	Healthy subjects (*N* = 127)	Patients (*N* = 151)	*p*
	*N* (%)	*N* (%)	
*GC1S*	270 (53.15)	306 (50.66)	>0.05
*GC1F*	138 (27.17)	32 (5.30)	≤0.005
*GC2*	72 (14.17)	94 (15.56)	>0.05
*GC3*	28 (5.51)	172 (28.48)	≤0.005
Total number of alleles	508	604	

*N*, number of subjects; *N* (%), number of subjects (percentage); >, greater than; ≤, less than equal to. *p*-values were generated using the Pearson chi-square test.

### Gc phenotypes

In the studied populations of controls and patients, different VDBP/GC alleles (*GCa*) resulted into nine Gc phenotypes. The comparative accounts of these phenotypes are presented in Table 3. The most common phenotype was Gc1S/Gc1S in both controls (*N* = 43, 33.6%) and patients (*N* = 49, 32.5%), followed by Gc1S/Gc2 + Gc1F/Gc3 [controls (*N* = 31, 24.4%) vs. patients (*N* = 30, 19.9%)], without any significant differences among controls and patients. However, significant differences were observed in the distribution of other phenotypes among controls and patients, i.e., the frequency of Gc1S/Gc1F phenotype was higher in controls than in patients (controls *N* = 33, 26.0% vs. patients: *N* = 1, 0.7%, *p*^a^ < 0.001), Gc2/Gc1F was present only in controls (controls *N* = 14, 11.0% vs. patients *N* = 0, *p*^b^ < 0.001); whereas, Gc1S/Gc3 (patients: *N* = 39, 25.9% vs. controls: *N* = 0, *p*^a^ < 0.001) and Gc3/Gc3 (controls: *N* = 0, 0.0% vs. patients: *N* = 9, 6.0%, *p*^a^ < 0.001) were found only in patients (Table 3). The phenotype Gc1F/Gc1F was present only in controls (*N* = 3, 2.4%) and not in patients (*N* = 0, 0.0%, *p*^b^ 0.094), but the number was too low to give rise significant difference (Table 3). In Table 3 *p*-values were generated using the Pearson chi-square test (*p*^a^) Fisher’s Exact test (*p*^b^).

**Table 3:** Distribution of Gc phenotypes among the healthy controls and MS patients.

	Healthy subjects	MS patients	*p*
	*N* (%)	*N* (%)	
Gc phenotypes	*N* = 127	*N* = 151	
Gc1S/Gc1S	43 (33.9)	49 (32.5)	0.804^a^
Gc1S/Gc2/Gc1F/Gc3	31 (24.4)	30 (19.9)	0.468^a^
Gc1S/Gc3	0 (0.0)	39 (25.8)	<0.001^a^
Gc1S/Gc1F	33 (26.0)	1 (0.7)	<0.001^a^
Gc2/Gc1F	14 (11.0)	0 (0.0)	<0.001^b^
Gc2/G3	0 (0.0)	14 (9.3)	<0.001^a^
Gc2/Gc2	3 (2.4)	9 (6.0)	0.141^a^
Gc3/Gc3	0 (0.8)	9 (6.0)	0.024^b^
Gc1F/Gc1F	3 (2.4)	0 (0.0)	0.094^b^
Gc phenotypes, 5 groups			
Gc1S/Gc1S	43 (33.9)	49 (32.5)	0.799^a^
Gc1S/Gc3 + Gc2/Gc3 + Gc3/Gc33	0 (0.0)	62 (41.1)	<0.001^a^
Gc1S/Gc2/Gc1F/Gc3	31 (24.4)	30 (19.9)	0.466^a^
Gc1F/Gc1F + Gc1S/Gc1F + Gc2/Gc1F	50 (39.4)	1 (0.7)	<0.001^a^
Gc2/Gc2	3 (2.4)	9 (6.0)	0.235^a^

*N*, number of subjects; *N* (%), number of subjects (percentage); <, less than. *p*-values were generated using ^a^ = Pearson chi-square test and ^b^ = Fisher’s exact test.

In a somatic cell (diploid cell), each chromosome is present in two copies and each *GCa* can be present in homozygous or heterozygous associations. On the basis of homozygous and heterozygous distributions, we clustered the Gc phenotypes into 5 phenotypic groups, which are Gc1S/Gc1S (homozygous at both loci without mutations, wild type), Gc1S/Gc3 + Gc2/Gc3 + Gc3/Gc3 (heterozygous at one locus and homozygous at the other), Gc1S/Gc2 + Gc1F/Gc3 (heterozygous at both loci), Gc1F/Gc1F + Gc1S/Gc1F + Gc2/Gc1F (homozygous at one locus and heterozygous at the other) and Gc2/Gc2 (homozygous at both loci with mutations) (Table 4). It was found that Gc1S/Gc1S was predominant with almost equal frequency in both the groups (healthy controls: *N* = 43, 33.9% vs. patients: *N* = 49, 32.5%, *p* = 0.799), followed by heterozygous Gc1S/Gc2/Gc1F/Gc3 (healthy controls: *N* = 31, 24.4% vs. patients: *N* = 30, 19.9%, *p* = 0.466). However, other Gc3 containing phenotypes (Gc1S/Gc3 + GC2/Gc3 + Gc3/Gc3) were present only in patients but not in controls (healthy controls: *N* = 0, 0.0% vs. patients: *N* = 62, 41.1%; *p* < 0.001) and the presence of Gc1F containing phenotypes (Gc1F/Gc1F + Gc1S/Gc1F + Gc2/Gc1F) was significantly higher in controls than in patients (healthy controls: *N* = 50, 39.4% vs. patients: *N* = 1, 0.7%; *p* < 0.001). The distribution of mutant homozygous phenotype Gc2/Gc2 did not significantly differ in patients and controls (Table 4). In Table 4, *p*-values were generated using the Mann–Whitney test.

**Table 4:** Concentration of 25(OH)vitamin D and VDBP in serum.

	All *N* = 278	Healthy subjects *N* = 127	MS patients *N* = 151	*p*
	Median^*^	Median^*^	Median^*^	
Total 25(OH)vitamin D (nmol/L)	28.0 (17.7–49.3)	28.1 (17.9–47.6)	28.0 (17.1–50.6)	0.848
Vitamin D binding protein (μg/mL)	200.3 (132.5–258.3)	235.8 (152.5–286.7)	158.8 (115.8–238.4)	<0.001

<, less than; median^*^, median (25th–75th percentile); *p*-values were generated using Mann–Whitney test.

### Serum 25(OH)vitamin D and VDBP levels in the study groups

In the studied subjects, hypovitaminosis D (<50 nmol/L) was common in both patients and controls and they were equally deficient in total 25(OH)vitamin D (nmol/L) level [controls: 28.1 (17.9–47.6) vs. patients: 28.0 (17.1–50.6); *p* = 0.848]; total 25(OH)vitamin D (nmol/L) represented by median (25th–75th percentile) (Table 5). However, the serum level of VDBP (μg/mL) was significantly higher in controls than in patients [controls: 235.8 (152.5–286.7) vs. patients: 158.8 (115.8–238.4); *p* = <0.001] represented by median (25th–75th percentile) (Table 5); *p*-values were generated using the Mann–Whitney test.

**Table 5:** The serum concentrations of total 25(OH)vitamin D and VDBP in relation to the absence or presence or of various *GC* alleles.

GC allele	Total 25(OH)vitamin D (nmol/L)				VDBP (μg/mL)			
	Healthy subjects		MS patients		Healthy subjects		MS patients	
	Median^*^	*p*	Median^*^	*p*	Median^*^	*p*	Median^*^	*p*-value
*GC1F*								
Absent	27.6 (17.7–38.2)	0.372	30.2 (17.0–50.9)	0.559	278.4 (216.8–363.0)	<0.001	154.6 (114.8–231.7)	0.411
Present	28.6 (17.9–50.7)		24.7 (21.1–39.4)		198.4 (147.2–259.7)		203.7 (140.9–258.7)	
*GC1S*								
Absent	33.6 (18.6–57.4)	0.537	29.8 (22.0–53.3)	0.338	149.4 (117.3–246.6)	0.002	146.7 (105.3–199.5)	0.001
Present	28.0 (17.8–41.4)		27.3 (16.3–49.5)		244.7 (174.5–290.6)		173.8 (122.9–241.4)	
*GC2*								
Absent	26.8 (16.9–38.4)	0.048	30.2 (16.6–51.0)	0.710	237.0 (169.7–303.3)	0.078	154.6 (114.8–228.4)	0.923
Present	32.2 (19.7–57.4)		26.2 (21.1–46.9)		216.6 (142.0–270.1)		189.2 (125.0–254.6)	
*GC3*								
Absent	26.8 (16.9–40.6)	0.025	35.3 (17.1–59.7)	0.156	223.4 (147.5–286.7)	0.603	189.5 (118.9–247.3)	0.026
Present	35.9 (23.7–64.4)		26.8 (17.2–42.5)		244.7 (191.0–280.8)		151.8 (115.8–227.7)	

Median^*^, median (25th–75th percentile); *p*-values were generated using the Mann–Whitney test.

### Serum 25(OH)vitamin D and VDBP levels in relation to *GCa*

The analysis of vitamin D and VDBP levels in sera of patients and controls showed that all of them were vitamin D deficient (<50 nmol/L) in relation to the presence or absence of all the four *GCa* (Table 6). The role of each *GCa* on serum 25(OH)vitamin D and VDBP concentrations was analyzed by comparing their concentrations in the presence and absence of specific *GCa* in the study cohort (Table 6). These analyses revealed no association between the presence or absence of any of the four *GCa* and the total 25(OH)vitamin D levels in patients and controls. However, the presence of *GC1S* allele was linked to higher levels of VDBP in both controls (*p* < 0.002) and patients (*p* < 0.001). Despite this, patients having the *GC1S* allele exhibited lower VDBP concentrations compared to controls. On the other hand, the presence of *GC1F* allele was associated with significantly lower level of VDBP in controls only (*p* < 0.001), and the presence of *GC3* allele was significantly associated with lower level of VDBP in patients only (*p* < 0.026) (Table 6). There was no significant difference in the concentration of VDBP level in controls.

**Table 6:** The serum concentrations of total 25(OH)vitamin D (nmol/L) and VDBP (μg/mL) in relation to various Gc phenotypes.

	Healthy subjects		Patients			Healthy subjects		Patients		
	Total 25(OH)vitamin D		Total 25(OH)vitamin D			VDBP		VDBP		
	Median *	*p* ^a^	Median^*^	*p* ^b^	*p* ^c^	Median *	*p* ^a^	Median^*^	*p* ^b^	*p* ^c^
Gc phenotypes										
Gc1S/Gc1S	27.6 (17.5–38.8)	0.136	35.1 (16.7–59.6)	0.398	0.320	286.7 (214.2–371.0)	<0.001	183.9 (106.0–245.8)	0.043	0.000
Gc1S/Gc2/Gc1F/Gc3	33.7 (22.0–58.0)		24.7 (19.8–39.1)		0.097	244.7 (174.9–285.3)		205.3 (136.9–258.7)		0.048
Gc1S/Gc3	—		28.5 (14.8–44.2)		—	—		151.2 (115.8–225.7)		—
Gc1S/Gc1F	25.2 (16.6–30.1)		92.8 (92.8–92.8)		0.092	194.4 (149.8–240.5)		203.7 (203.7–203.7)		0.834
Gc2/Gc2	22.6 (11.8–27.7)		48.2 (20.3–56.2)		0.967	251.0 (246.8–255.2)		211.4 (146.6–276.7)	0.483
Gc2/Gc1F	43.1 (17.6–60.9)	—	—	137.6 (99.9–161.9)	—	—
Gc2/Gc3	—	29.8 (20.4–45.4)	—	—	149.2 (96.5–197.9)	—
Gc3/Gc3	—	27.5 (24.2–55.8)	—	—	114.7 (76.3–139.0)	—
Gc1F/Gc1F	33.6 (10.1–49.1)	—	—	237.0 (108.5–306.5)	—		—
GC phenotypes 5 groups										
Gc1S/Gc1S	27.6 (17.5–38.8)	0.112	35.1 (16.7–59.6)	0.323	0.320	286.7 (214.2–371.0)	<0.001	183.9 (106.0–245.8)	0.089	0.000
Gc1S/Gc3 + Gc3/Gc3 + Gc2/Gc3	—	28.2 (16.0–44.5)	0.124	—	146.8 (114.8–212.5)	—
Gc1S/Gc2/Gc1F/Gc3	33.7 (22.0–58.0)	24.7 (19.8–39.0)	0.097	244.7 (174.9–285.3)	205.3 (136.9–258.7)	0.048
Gc1F/Gc1F + Gc1S/Gc1F + Gc2/Gc1F	26.2 (16.6–44.0)	92.8 (92.8–92.8)	0.089	182.1 (137.1–236.4)	203.7 (203.7–203.7)	0.652
Gc2/Gc2	22.6 (11.8–27.7)	48.2 (20.3–56.2)	0.405	251.0 (246.8–255.2)	211.4 (146.6–276.7)	0.480

Median^*^, median (25th–75th percentile); <, less than. *p*^a^ = Significance among different genotypes in control subjects. *p*^b^ = Significance among different genotypes in patients. *p*^c^ = Significance between control and patients. *p*^a^ and *p*^b^ values were generated using the Kruskal–Wallis test and *p*^c^ were generated using the Mann–Whitney test.

### Serum 25(OH)vitamin D and VDBP levels in relation to Gc phenotypes

The analysis of 25(OH)vitamin D and VDBP levels in sera of patients and controls showed that all of them were vitamin D deficient (<50 nmol/L) or insufficient (50–75 nmol/L) in relation to various Gc phenotypes, except one patient, having Gc1S/Gc1F phenotype, was associated with sufficient level of 25(OH)vitamin D, i.e., >75 nmol/L (Table 6). This was possibly an outlier than a norm. The VDBP level in healthy controls were significantly lower in subjects with phenotypes Gc1F/Gc1F + Gc2/Gc1F + Gc1S/Gc1F (Table 6). The comparisons of VDBP levels showed that the patients with Gc1S/Gc1s and Gc1S/Gc2/Gc1F/Gc3 phenotypes had significantly lower levels than controls (Table 6).

## Discussion

This study was performed to determine the association of VDBP/Gc genotypes and phenotypes with the concentrations of 25(OH)vitamin D and VDBP in healthy subjects and MS patients of Kuwaiti nationality. The results showed the presence of four alleles in Kuwaiti population, i.e., *GC1S*, *GC1F*, *GC2*, and *GC3*. Among these, *GC1S* and *GC2* were present in both homozygous and heterozygous combinations in healthy subjects and MS patients, while *GC3* was present in both homozygous and heterozygous combinations in MS patients but it was present only in heterozygous combinations in healthy subjects. In contrast, *GC1F* was present in both homozygous and heterozygous combinations in healthy subjects but it was present only in heterozygous combinations in MS patients. Most of the previous studies have shown the existence of only three alleles of *GC*, i.e., *GC1S*, *GC1F*, and *GC2* in various populations around the world (37–39), including the Middle East (40–42). The fourth allele *GC3* was reported only in a single study conducted in a young and healthy Lebanese population (43). Recently, we reported the presence of the rare allele *GC3* in Kuwaiti subjects (21). However, as compared to our study in which *GC3* was present only in a heterozygous combination in healthy subjects, in the Lebanese study, *GC3* was present in both homozygous and heterozygous combinations in healthy subjects. It is worth mentioning here that the Lebanese study did not include any patient group to compare the frequency *GC3* with the healthy subjects (43).

The analyses of our results with respect to *GCa* and association with MS patients and healthy subjects revealed that the frequency of *GC1F* was significantly higher in healthy controls and *GC3* was significantly higher in MS patients, whereas there were no significant differences for the frequencies of *GC1S*, and *GC2* alleles in the healthy controls and MS patients. These results suggest that *GC1F* may be protective and *GC3* pathogenic for MS. Previous studies conducted in Japan, Canada and Italy did not include *GC3* in their experiments and *GC1F* was not associated with protection or pathogenesis against MS (13, 24, 25, 44). The results obtained in our study may be population-specific because population-specific associations for *GCa* have been reported in other diseases, e.g., chronic obstructive pulmonary disease (40, 45). However, the association of *GC1F* with protection has been reported in other diseases, like cancer (46).

We observed that the homozygous alleles (TT/CC) at both loci (rs4077/rs4588) leading to the phenotype Gc1F/Gc1F and homozygous alleles at one locus and heterozygous alleles at the other locus (GT/CC or TT/CA) leading to phenotypes Gc1S/Gc1F or Gc2/Gc1F were expressed at higher frequency in controls than patients. Whereas *GC3* homozygous alleles (GG/AA) at both loci (rs4077/rs4588) leading to phenotype Gc3/Gc3 and homozygous alleles at one locus and heterozygous alleles at the other locus (GG/CA and GT/AA) leading to phenotypes Gc1S/Gc3 and Gc2/Gc3 were expressed at higher frequency in patients than controls. However, the phenotype containing both Gc1F and Gc3 in a single individual, i.e., Gc1S/Gc2/Gc1F/Gc3 due to heterozygous alleles GT/CA at both loci (rs4077 and rs4588), was expressed in equal frequencies in both MS patients and healthy subjects. These results suggest that the presence of the Gc3 and Gc1F in the same individual appeared to nullify their independent pathogenic and protective effects.

Our study indicates that both groups of subjects were deficient in 25(OH)vitamin D levels in blood and that there were no significant differences in the 25(OH)vitamin D levels in the two groups. These results are compatible with a previous report from Kuwait showing vitamin D deficiency in both healthy subjects and MS patients (47). Furthermore, no differences in the vitamin D level among healthy subjects and MS patients have been reported from other parts of the world as well (48), and no significant association between high-dose vitamin D treatment and risk of MS relapses was found in a meta-analysis published in 2013 (49). However, an increasing volume of work suggests that lower levels of serum vitamin D are associated with an increased risk of MS and a more severe disease course (50). Furthermore, recent study has suggested that vitamin D supplementation may have a therapeutic role in the treatment of MS and improvements in disease measures may be more apparent in those with lower baseline vitamin D levels (51).

Our finding that GC3 and GC1F are associated with the incidence of MS is in line with Gezmis’s et al. (52) observation that rs4588 variations are associated with an increased risk of developing MS. However, since all our patients and controls had vitamin D deficiency, we were unable to find a link between the two conditions.

We noted that VDBP concentrations were significantly higher in controls than in patients. As VDBP binds vitamin D, the higher concentrations of VDBP may be associated with lower concentrations of vitamin D in serum (53). However, our results did not support this hypothesis as vitamin D concentrations were similar and insufficient in both controls and MS patients. Our results in this and a previous study indicate that lower VDBP levels could be associated with MS pathogenesis (26). Somewhat similar results have been reported by Qin et al. (23) showing that lower concentrations of VDBP were found in the cerebrospinal fluid of relapsing and remitting MS patients during acute relapse compared to patients with other neurological diseases. However, some other studies have either shown a lack of association for VDBP level in MS patients (49, 54), or presence of an increased concentration of it in MS patients (55, 56). The difference in our study and the previous studies could be due to the variations in the studied populations.

In both groups, no significant difference in 25(OH)vitamin D levels was noted among healthy subjects and MS patients carrying various Gc phenotypes. However, we observed a protective role of Gc1F against MS risk in our cohort. Given that higher levels of 25(OH)vitamin D were associated with a lower risk of MS (57), our result aligned with those of Agnello et al. (44) who reported that patients who carried Gc1F showed higher plasma levels of Vitamin D. In this regard, it is worth to acknowledge that vitamin D is known to have some important immunomodulatory roles in the activation of T cells and their homeostasis (58), and the low VDBP in the cerebrospinal fluid during MS relapses might be mediated by vitamin D deficiency related activation of cellular immunity mediated by T cells. VDBP also regulates the availability of vitamin D and its response to antimicrobial monocytes (59). Hence, it is hypothesized that changes in VDBP levels are capable of favoring infections that are associated with relapses in MS patients (59).

Furthermore, the VDBP concentrations were significantly less in the serum of MS patients than controls with respect to the homozygous Gc phenotype Gc1S/Gc1S and heterozygous Gc phenotype Gc1S/Gc2/Gc1F/Gc3 (Table 6). However, as discussed above, these phenotypes are not associated with MS risk in Kuwaiti population. The concentrations of VDBP among MS patients and healthy subjects could not be due to phenotypes associated with MS pathogenesis (Gc1S/Gc3 + Gc3/Gc3 + Gc2/Gc3) and protection (Gc1F/Gc1F + Gc1S/Gc1F + Gc2/Gc1F) because the phenotypes Gc1S/Gc3 + Gc3/Gc3 + Gc2/Gc3 were absent in healthy subjects and only one MS patient had the phenotypes Gc1F/Gc1F + Gc1S/Gc1F + Gc2/Gc1F. We could not find any published study in the English literature to compare the levels of VDBP in relation to VDBP/Gc phenotypes in MS patients.

One important limitation of this study is that like any other case–control study the potential for recall bias cannot be ruled out. Though, an appropriate control group is recruited in this study, the expression of biochemical variables can be influenced by environmental and sociocultural practice like available solar intensity, dietary habit, shrouded dressing, and exposure to sunlight to name a few.

The results of this study have the potential to stimulate researchers from other parts of the world to look for such associations in their patients. Thus, our results, in addition to being relevant for Kuwait and the Gulf Region, may also have global implications.

## Conclusion

In this study, we identified three common *VDBP*/*GC* subtypes (*GC1S*, *GC1F*, *GC2*) previously reported in the literature and *the novel subtypes GC3* in both MS patients and healthy subjects. The GC3 was not previously reported in MS patients. The subtype *GC1F* was associated with protection and *GC3* had association with pathogenesis in MS. However, when both *GC1F* and *GC3* were present in the same individual, they appear to nullify their independent effect. Vitamin D levels were deficient in both patients and controls and there were no associations with the vitamin D level and different *GCa* alleles and phenotypes. On the other hand, VDBP concentrations were significantly low in MS patients than healthy subjects.

To the best of our knowledge, this is the first study showing the association of varying *VDBP/GC* genotypes and phenotypes in protection as well as pathogenesis of MS in Kuwaiti nationals. Moreover, our study has shown that there is generalized hypovitaminosis D in Kuwaiti population, which reinforces the idea that effective measures should be taken to overcome the problem in public. Furthermore, the association of *VDBP/GC* genotypes and phenotypes with MS in Kuwaiti patients opens the door for similar studies in the Gulf Region.

## Data Availability

The raw data supporting the conclusions of this article will be made available by the authors, without undue reservation.

## Glossary

VDBP: Vitamin D binding protein
Gc: Group-specific component
*GCa*: VDBP/GC alleles
*GC1F*: Group-specific component fast genotype
*GC1S*: Group-specific component slow genotype
*GC2*: Group-specific component genotype 2
*GC3*: Group-specific component genotype 3
Gc1F: Group-specific component fast phenotype
Gc1S: Group-specific component slow phenotype
Gc2: Group-specific component phenotype 2
Gc3: Group-specific component phenotype 3
MS: Multiple sclerosis
25(OH)vitamin D: 25 hydroxyvitamin D
HLA: Human leukocyte antigen
APo E: Apolipoprotein E
25(OH) D3: 25 hydroxyvitamin D3
SNPs: Single nucleotide polymorphisms
BMI: Body mass index
EIA: Enzyme immunoassay
NCBI: National Centre for Biotechnology Information
F primer: Forward primer
R primer: Reverse primer
SPSS: Statistical Package for Social Sciences
Mean ± SD: Mean ± standard deviation
≤: Less than equal to
*N*: Number of subjects
>: Greater than
*N* (%): Number of subjects (percentage)
